# Emerging zoonotic risks: whole-genome sequencing reveals antimicrobial resistance and genomic diversity in *Providencia stuartii* isolated from broiler chickens in Noakhali, Bangladesh

**DOI:** 10.1016/j.psj.2026.106602

**Published:** 2026-02-10

**Authors:** Israt Jahan Asha, Shipan Das Gupta, Md. Adnan Munim, Nurun Nahar Akter, Saheda Tamanna, Anisur Rahman, Al Imran, Shuvo Chandra Das, Md. Murad Hossain, Mohammed Mafizul Islam, Dhirendra Nath Barman

**Affiliations:** a Department of Biotechnology and Genetic Engineering, Noakhali Science and Technology University, Noakhali 3814, Bangladesh; b Department of Computer Science and Engineering, American International University, Bangladesh

**Keywords:** Multi-drug resistance, Pathogenicity, *Providencia stuartii*, Poultry chicken, Whole-genome sequencing, Zoonotic transmission

## Abstract

*Providencia stuartii* is emerging as an Extensively Drug-Resistant (XDR) pathogen commonly found in animals, insects, and in burned and immunocompromised conditions. The misuse of antibiotics in poultry feed causes the emergence of XDR bacteria in the poultry industry. The knowledge of zoonotic transmissibility of poultry-derived *P. stuartii* remains elusive in Noakhali, Bangladesh. Poultry fecal and rectal swab samples were collected from selected farms in Noakhali, Bangladesh. Bacterial isolation and identification were performed using MacConkey agar, biochemical tests, and *16S* rRNA Sanger sequencing. Antimicrobial susceptibility was assessed by the Kirby-Bauer disk diffusion method, and isolates with high multiple antibiotic resistance (MAR) indices were selected for whole-genome sequencing (WGS). Quality control, genome assembly, annotation, gene identification, pan-genome analysis, pathogenicity profiling, and comparative proteome analyses were subsequently conducted. Antibiogram analysis showed that ps_nstu_001 and ps_nstu_002 were resistant to 17 and 13 tested antibiotics, respectively. Furthermore, whole-genome sequencing revealed that both strains harbored resistance determinants to aminoglycosides, tetracyclines, sulfonamides, cephalosporins, β-lactams, and carbapenems. Additionally, mobile genetic elements (MGEs) and plasmids were identified, which represent the horizontal gene transfer capability. Moreover, pangenome analysis revealed ongoing gene acquisition and substantial genomic diversity among the isolates. The isolate ps_nstu_001 was identified as a putative human pathogen and clustered closely with a clinical strain isolated in the United States. In contrast, ps_nstu_002 was predicted to be a non-human pathogen; however, it exhibited a clear evolutionary relationship with a clinical isolate obtained from a diarrheal patient in Bangladesh, suggesting potential pathogenic relevance. Global pathogenic potential of the studied strains and key proteomic similarities between pathogenic and non-pathogenic strains revealed by pathogenicity profiling and proteome comparison. To conclude, these XDR isolates indicate the potential for zoonotic transmission and the spread of resistant genes to other animals, posing a significant public health risk.

## Introduction

Antimicrobial resistance has emerged as a major public health challenge worldwide, with food-producing animals playing a critical role in amplifying and disseminating resistant bacteria (Sweileh, 2021). The poultry sector is of particular concern due to intensive farming practices, high stocking densities, and the widespread use of antimicrobials for growth promotion, prophylaxis, and disease control (Hedman et al., 2020). In low- and middle-income countries such as Bangladesh, limited regulatory oversight and easy access to antibiotics have further accelerated the emergence of multi-drug resistant (MDR) and extensively drug-resistant bacterial pathogens within poultry production systems. Poultry-associated bacteria can act as reservoirs of antimicrobial resistance genes that spread through direct contact, food consumption, environmental contamination, and horizontal gene transfer.

Several studies from Bangladesh have documented high resistance rates in Enterobacteriaceae isolated from broiler chickens, including resistance to critically important antimicrobials such as third-generation cephalosporins, fluoroquinolones, carbapenems, and colistin (Hossain et al., 2021; Islam et al., 2023). The detection of plasmid-mediated resistance genes and MGEs in poultry isolates highlights the potential for rapid dissemination of resistance across bacterial species and ecological niches. Monitoring antimicrobial resistance in the poultry sector is therefore essential, not only to safeguard animal health but also to mitigate zoonotic transmission to humans. Because, zoonotic pathogens originating from poultry have been increasingly implicated in human infections, particularly among immunocompromised individuals and hospital settings (Mellata, 2013; El-ghany, 2021). Additionally, opportunistic pathogens such as *P. stuartii*, traditionally associated with urinary tract infections, septicemia, and burn wounds in humans, have recently been reported from animal and environmental sources, raising concerns about their zoonotic potential (Nahar et al., 2016; Mondol et al., 2024). Consequently, Bangladesh represents a high-risk setting for the emergence and spread of zoonotic antimicrobial resistance due to dense human and poultry populations, close human-animal interactions, and inadequate antimicrobial stewardship (Khan et al., 2020).

*P. stuartii* primarily cause respiratory and urinary tract infections, and is associated with biofilm formation in catheterized patients. It is also responsible for significant outbreaks in hospital environments (Capitani et al., 2023). It has been identified with novel transposons carrying extended-spectrum beta-lactamase (ESBL) genes, conferring resistance to multiple beta-lactam antibiotics. The presence of extensively drug-resistant *P. stuartii* in poultry would represent a significant One Health threat, as such strains may enter the human population through the food chain or environmental exposure. Liu et al. (2020) reported that 76 isolates of *P. stuartii* expressed ESBLs, with 92.1% exhibiting multi-drug or extensive drug resistance (Liu et al., 2020). MDR *P. stuartii* in chicken droppings may facilitate its spread by person-to-person transmission (Guidone et al., 2023) and via the human-to-animal interface. They may impose a threat of cross-resistance to other pathogens, leading to reduced antibiotic effectiveness in curing or preventing infections (Nahar et al., 2016). Recently, numerous *P. stuartii* strains with *bla*NDM have been reported, including those carrying *bla*NDM on *IncC*-type plasmids (Mc Gann et al., 2012; Manageiro et al., 2015; Abdallah and Balshi, 2018). These findings highlight the significant role of *P. stuartii* in the spread of AMR genes.

The Noakhali region, in particular, has been identified as a hotspot for MDR Gram-negative bacteria in poultry, with resistance profiles comparable to those reported in clinical settings (Akter et al., 2026: Munim et al., 2024). Despite this growing body of evidence, genomic-level investigations exploring the zoonotic transmissibility, pathogenic potential, and evolutionary dynamics of poultry-derived *P. stuartii* remain limited. In this context, WGS offers a powerful approach to elucidate resistance mechanisms, MGEs, pan-genome dynamics, and phylogenetic relationships between animal and human isolates. Comprehensive genomic surveillance of poultry-derived *P. stuartii* is therefore critical to understanding its role in the dissemination of antimicrobial resistance and to assessing its potential contribution to zoonotic infections in Bangladesh. Recent evidence demonstrates that the Noakhali region serves as a significant reservoir of MDR bacteria within Bangladesh’s poultry production system. A comprehensive investigation of broiler and layer chickens from Noakhali revealed widespread resistance among *Escherichia coli, Klebsiella pneumoniae, Providencia* spp., and *Aeromonas* spp., with all isolates exhibiting multiple antibiotic resistance indices exceeding the threshold for high-risk MDR classification. Critically important antimicrobials, including colistin, fluoroquinolones, and third-generation cephalosporins, showed markedly reduced efficacy, while plasmid-mediated resistance genes such as *blaTEM, blaSHV, tet, sul*, and *mcr-1* were frequently detected. These findings confirm that poultry in Noakhali act as a stable environmental and zoonotic reservoir of anti-microbial resistant (AMR) determinants, reinforcing the region’s relevance for resistance surveillance and molecular characterization studies (Munim et al., 2024). Some related studies demonstrated the extensive dissemination of carbapenemase-producing and MDR pathogens across Bangladeshi poultry systems. A high prevalence of MDR *K. pneumoniae* and *E. coli* harboring clinically significant β-lactamase and carbapenemase genes, alongside strong biofilm-forming capacity that enhances persistence and horizontal gene transfer (Saha et al., 2023; Uddin et al., 2025). Importantly, Saha et al. (2023) highlighted co-resistance to heavy metals, indicating environmental co-selection pressure that sustains resistance even in the absence of antimicrobial exposure.

From 2017 to 2022, the government of Bangladesh developed a National Action Plan to control antibiotic-resistant and MDR bacteria across the animal, environmental, and human sectors (Hoque et al., 2020). Additionally, to understand and monitor such resistance patterns, WGS is used to analyze bacterial pathogens, providing insights into antibiotic resistance, molecular epidemiology, and virulence, and is increasingly applied in research and clinical diagnostics (Quainoo et al., 2017). Consequently, there are significant knowledge gaps regarding numerous potential zoonotic pathogens that may be transmitted through the poultry industry (Dahms et al., 2014). Notably, no WGS data on *P. stuartii* isolated from poultry in Noakhali, Bangladesh, or elsewhere in Bangladesh have been published before. Besides, genomic characterization of *P. stuartii* including resistance genes, virulence genes, plasmids, and zoonotic potentials, was also not recorded.

In line with these actions, this study aims to address a critical public health concern for the poultry industry and humans by identifying and characterizing *P. stuartii* isolates (ps_nstu_001 and ps_nstu_002) from poultry in Noakhali, Bangladesh, with the following objectives: (i) isolation and identification of *P. stuartii* isolates by biochemical and molecular approaches; (ii) evaluation of resistance patterns of MDR *P. stuartii* isolates of poultry chickens; (iii) identification of antibiotic-resistance genes (ARGs) and their genetic relatedness; (iv) examination of the possible transmission capabilities of antibiotic resistance and virulence genes between poultry-derived *P. stuartii* isolates and clinical MDR strains. This study highlights the dynamics of antibiotic resistance in poultry farming environments and the related public health concerns in Noakhali, Bangladesh. It will be helpful to guide strategies for the wise use of antibiotics in the poultry industry.

## MATERIALS AND METHODS

### Sample Collection and Identification

Four distinct small-scale commercial broiler farms situated in Noakhali Sadar were selected for sampling. Comprehensive data on the chickens, including their age, body weight, feeding regimen, health status, and origin, were recorded (Supplementary Table S1). Fecal samples and rectal swabs were collected under sterile conditions to minimize contamination during sampling and transport. Each specimen was sealed in sterile conical centrifuge tubes and Petri plates using parafilm, ensuring proper labeling and biosafety. In total, 160 bacterial isolates from 12 samples were collected, from six broiler and six layer chickens. From the overall 160 isolates, ten *P. stuarti* isolates were obtained solely from broiler chicken samples.

Fecal and rectal swab specimens were immediately diluted tenfold by mixing one ml of the sample with nine ml of sterile saline. This dilution process was repeated five times. Afterward, 50 µl of the diluted samples were spread onto the MacConkey agar and Eosin Methylene Blue (EMB) agar media and incubated at 37 °C for 18-22 h. Primarily, pure colonies were obtained based on colony morphology by subculturing the isolates on EMB agar plates at 37 °C for 18-22 h. *P. stuartii* isolates were presumptively identified using six standard biochemical tests, including lactose fermentation, triple sugar iron, motility, urease, indole, and oxidase tests, following the protocols outlined in Bergey’s Manual of Determinative Bacteriology (Bergey, 1957).

For molecular identification, DNA was extracted and used to amplify the *16S* rRNA gene via polymerase chain reaction using universal primers *27F* (5′-AGAGTTTGATCCTGGCTCAG-3′) and *1492R* (5′-GGTTACCTTGTTACGACTT-3′) as described in a previous study (Munim et al., 2024). The obtained *16S* rRNA amplicons were sequenced commercially at the National Institute of Biotechnology, Bangladesh, through Sanger di-deoxy sequencing. Sequenced data were visualized using SnapGene Viewer 6.0.5 (https://www.snapgene.com/snapgene-viewer) and analyzed using the Nucleotide BLAST (BLASTn) tool with default parameters for species-level identification (Altschul et al., 1990). Only high-quality reads with clear chromatogram peaks and no ambiguous bases were included for downstream analysis; sequences with poor quality scores, ambiguous bases (N), or <800 bp length were excluded.

### Phenotypic Antimicrobial Susceptibility Test

Antimicrobial susceptibility of the collected ten *P. stuartii* isolates was assessed (Schiller et al., 2022), and the results were interpreted according to the Clinical and Laboratory Standards Institute guidelines 2018 (CLSI, 2018). A total of 17 antibiotics (Supplementary Table S2) on Mueller Hinton agar (Oxoid, UK) were used following the Kirby-Bauer disk diffusion method (Microbiology, 2016). Inoculum density (McFarland standard), incubation time, temperature, and disk placement were performed according to the above-mentioned protocol. Isolates exhibiting a MAR index greater than 0.2 were classified as MDR *P. stuartii* (Titilawo et al., 2015).

### Genomic Library Preparation and Whole-Genome Sequencing

Following screening for the highest MAR index among *P. stuartii* isolates, two were selected for whole-genome characterization. Genomic DNA was extracted using the QIAamp DNA Mini Kit (Qiagen) following the manufacturer’s protocol (Smith, 2011). The extracted DNA underwent rigorous quality control assessments, including the determination of concentration and purity using a Nanodrop spectrophotometer (Thermo Fisher Scientific, USA). To prepare the sequencing libraries, a paired-end library was generated for each isolate using the NEBNext® Ultra™ II FS DNA Library Prep Kit (New England Biolabs) (Gautreau, 2023). The integrity and fragment size distribution of the constructed libraries were assessed using AMPure XP beads (Beckman Coulter Life Sciences), gel electrophoresis, and concentration quantification via a Qubit 4 fluorometer (Thermo Fisher Scientific, USA). Finally, Illumina NextSeq 500 system was used for sequencing, following the NextSeq v2.5 reagent kit (2 × 150 bp) protocol in the Child Health Research Foundation (CHRF) (https://chrfbd.org/) and by Illumina HiSeq in the International Centre for Diarrhoeal Disease Research, Bangladesh (ICDDRB) (https://www.icddrb.org/).

### Rigorous Quality Control Assessments, Genomic Assembly, Annotation, and Species Identification

The raw sequenced file in FASTQ format underwent quality assessment using FastQC (v0.11) to evaluate read quality (Leggett et al., 2013). Raw sequencing reads were mapped to the reference genome using BWA with default parameters (Li and Durbin, 2009). Alignment files were processed using SAMtools for format conversion, sorting, and indexing. Per-base sequencing depth was calculated using samtools depth, and coverage breadth (%) was defined as the proportion of genomic positions covered by at least one read. To ensure high-quality sequences, adapter sequences and low-quality base regions were removed using Trimmomatic (Bolger et al., 2014). After trimming the obtained Fastqc reports of both isolates are shown in Supplementary Table S3 and S4. Paired-end Illumina sequencing reads were quality-assessed and filtered using fastp v1.1.0 (Chen, 2025) with default parameters. Post-filtering statistics were used to estimate sequencing yield and base quality. Raw data volume was calculated from the total number of bases generated after filtering, while sequencing quality was assessed using the Q30 metric reported by Fastp. All values represent combined statistics from both paired-end reads. Following quality filtering, the high-fidelity reads were assembled *de novo* using Unicycler (Wick et al., 2017), reconstructing the genome sequences. The genome assembly quality was validated using BUSCO v5.2.2 (Manni et al., 2021) and Quast (Quality Assessment Software for Tools) (Gurevich et al., 2013). For taxonomic identification, a *k*-mer-based classification approach was employed using the Kraken2 (Lu et al., 2022). The genetic similarity of *P. stuartii* strain ps_nstu_001 was determined by comparing nucleotide similarity using ANI and Blastn with another clinically pathogenic isolate of *P. stuartii. P. stuartii* PRV00011 (NZ_JADSTB010000001.1), and *P. stuartii* YD789-2 (PGGX01000001.1), and *P. stuartii* strain ps_nstu_002 was determined by comparing with *P. stuartii* strain NIB002 (JAVRQF010000001.1) and *P. stuartii* strain uvzsr-PT_24_00014-A12-AR_24_00000940 (CAXOTU010000001.1).

To annotate the genes presence on both studied genomes, the assembled sequences were analyzed using multiple annotation pipelines, including Prokka (Seemann, 2014), RAST (Aziz et al., 2008), and the Prokaryotic Genome Annotation Pipeline of NCBI (Tatusova et al., 2016). In the RAST server annotation job, the following settings were configured: Domain set to *Bacteria*, Genetic Code 11 (Archaea and Bacteria), and Annotation scheme set to Classic RAST with RASTtk enabled.

### Genome Mapping and Multi-locus Sequence Typing (MLST)

Circular genome maps were generated using the Proksee server (Grant et al., 2023). After that, MLST was performed for both isolates using PubMLST (Jolley et al., 2018). The sequence type of each isolate was determined by MLST by comparing the contig.fasta with global databases for epidemiological insights.

### Investigation of Anti-microbial Resistant Genes and Mobile Genetic Elements

AMR genes were identified using the Comprehensive Antibiotic Resistance Database (CARD) through the Resistance Gene Identifier (RGI) tool (McArthur et al., 2013). Default parameters were applied, and genome assemblies were analyzed against CARD’s curated resistance gene models. Only perfect and strict matches were included in the analysis, while low-quality reads and loose hits were excluded. In addition, AMR Finder Plus 4.0 (Feldgarden et al., 2021) was used to detect and interpret genes conferring antimicrobial resistance. The AMR Finder Plus virtual environment was activated prior to analysis, and assembled contigs were screened. The –plus option in the command enabled detection of additional resistance, stress response, and virulence-associated genes. Default parameters were used, and results were analyzed for high-confidence hits relevant to Enterobacteriaceae.

MGEs and plasmids within the whole genomes of the isolates were analyzed using the MGE Finder tool (Johansson et al., 2021), which is available on the Center for Genomic Epidemiology server. Besides, Plasmids were analyzed using Plasmid Finder (Carattoli et al., 2014) and MOB-suite (Robertson and Nash, 2018). Assembled genome contigs (contigs.fasta) were analyzed using the Enterobacteriales database, and default parameters were used to separate plasmid-derived contigs from chromosomal sequences and to predict plasmid mobility, replicon type, and host range.

### Pangenome Analysis and Heaps Law Plot

To investigate the genomic diversity of our two studied *P. stuartii* isolates, we extracted 48 *Providencia spp.* whole genome sequences of different geographical origins, host from the NCBI database and employed in the Roary pangenome pipeline (Page et al., 2015). The data is given in Supplementary Table S6. Genome annotation files (GFF3) of these 50 isolates were generated using Prokka, applying consistent annotation parameters across all genomes. A closely related NCBI reference genome of *P. stuartii* was annotated using the same pipeline to ensure compatibility. The resulting GFF3 files were used as input for Roary to perform pangenome analysis. Roary was run with a BLASTp identity cutoff suitable for bacterial genomes, enabling core gene identification and multiple sequence alignment using MAFFT v7.490 (Katoh and Standley, 2013). The concatenated core gene alignment was used to construct a maximum-likelihood phylogenetic tree with FastTree (Price et al., 2009) under the GTR nucleotide substitution model. Finally, visualization of the pan-genome, including features such as pie charts, histograms, and a gene presence/absence matrix, was completed using Roary’s accompanying Matplotlib in Python v3.12.2-based plotting utilities. Heaps’ Law (Kaur et al., 2024) was performed using the gene_presence_absence.csv file generated by Roary. The cumulative number of genes was calculated by sequentially adding genomes in random order and recording the corresponding increase in the pangenome size. This process was repeated multiple times to reduce sampling bias, and the average pangenome growth curve was fitted to Heaps’ law to assess whether the pangenome is open or closed. The resulting curve was visualized using custom Python scripts.

### Whole Genome-based Phylogenetic Analysis and Pathogenicity Profiling

A comprehensive phylogenomic analysis was conducted on 50 publicly available *P. stuartii* genomes from the NCBI database, including the studied isolates, using whole genome sequences and a single nucleotide polymorphism (SNP)-based approach. The resulting multiple sequence alignment data, incorporating SNPs and deleted sites by Snippy (https://github.com/tseemann/snippy), were used to construct and infer a phylogenetic tree via FastTree. Following the phylogenomic analysis, a rooted phylogenetic tree was generated and visualized using MEGA 11 software to understand the evolutionary relationships among the isolates (Tamura et al., 2021). Among the 50 whole genome sequences analyzed, most were derived from clinical human isolates, with two from broilers (including our study isolate) and one from a fly. From this dataset, 30 isolates were selected for comparative pathogenicity profiling. Pathogenicity prediction for all isolates concerning human hosts was performed using the PathogenFinder v1.1 web-based tool from the Center for Genomic Epidemiology (Cosentino et al., 2013), with data analysis and visualization carried out in Python v3.12 using Matplotlib. To further investigate the correlation between genomic characteristics and pathogenicity profiles, ten representative genomes from both pathogenic and non-pathogenic categories were selected for whole proteome comparison using bidirectional BLASTp.

## Result

### Species identification and evaluation of phenotypic resistance to antimicrobial agents of P. stuartii isolates

A total of ten *P. stuartii* isolates were presumptively identified from six broiler samples based on their biochemical characteristics and confirmed at the molecular level by *16S* rRNA Sanger deoxy sequencing. The detailed biochemical characteristics of the isolates are presented in Table 1. To phenotypically assess MDR, antimicrobial susceptibility tests were performed. The antibiotic resistance profiles of the ten *P. stuartii* isolates are presented in Table 2. Among 17 different antibiotics tested across 12 different classes, only Aztreonam showed a high efficacy showing susceptibility in 70% of the *P. stuartii* isolates (Table 2). Gentamicin was effective against five out of ten isolates (50%). Additionally, six and seven isolates demonstrated intermediate effects to norfloxacin and ciprofloxacin, respectively. However, a major concern is that the *P. stuartii* isolates showed complete (100%) resistance to seven distinct classes of antibiotics, including penicillins, phenicols, tetracyclines, carbapenems, macrolides, polymyxins, and the sulfonamide/trimethoprim combination.

**Table 1:** Biochemical characteristics of isolated bacteria from broiler chickens.

				MIU			Citrate	Oxidase	Lactose	Organism
Slant	Butt	H_2_S	Gas	Motility	Indole	Urea				
A	A	N	P	N	N	P	P	N	P	*Providencia stuartii*

**Table 2:** Antibiotic-resistant pattern displayed by ten out of ten *P. stuartii* isolates from broiler chickens.

Antibiotic	Resistant % (isolate number)	Intermediate% (isolate number)	Sensitive % (isolate number)
Ampicillin AMP25	100 (10/10)	0	0
Amoxicillin- Clavulanic acid AMC30	90 (9/10)	10 (1/10)	0
Aztreonam AT30	20 (2/10)	10 (1/10)	70 (7/10)
Chloramphenicol C30	100 (10/10)	0	0
Ciprofloxacin CIP 5	30 (3/10)	70 (7/10)	0
Norfloxacin NX10	40 (4/10)	60 (6/10)	0
Cefotaxime CTX30	80 (8/10)	10 (1/10)	10 (1/10)
Cefoxitin CX30	100 (10/10)	0	0
Gentamicin GEN 10	40 (4/10)	10 (1/10)	50 (5/10)
Kanamycin K30	100 (10/10)	0	0
Tetracycline TE30	100 (10/10)	0	0
Imipenem IMP 10	90 (9/10)	10 (1/10)	0
Azithromycin AZM30	100 (10/10)	0	0
Erythromycin E15	100 (10/10)	0	0
Colistin CL10	100 (10/10)	0	0
Polymyxin B PB300	100 (10/10)	0	0
Co-Trimoxazole COT25/ Sulph trimithoprime	100 (10/10)	0	0

### Antibiotic Resistance Patterns and Multiple Antibiotic Resistant Index Analysis of P. stuartii Isolates

Based on antibiotic susceptibility testing, the *P. stuartii* isolates were categorized into nine distinct antimicrobial-resistant profiles, with MAR indices ranging from 0.64 to 1.0. The complete antibiotic resistance patterns displayed by all ten *P. stuartii* isolates from broiler chickens are presented in Table 3. Notably, nine out of ten *P. stuartii* isolates exhibited MAR indices greater than 0.7, while six isolates had MAR indices exceeding 0.8. These findings highlight a significant AMR burden in the poultry sector, posing a potential threat to public health in the surrounding community.

**Table 3:** Antibiotic-resistant profiles with MAR index value of 10 *P. stuartii* isolates from broiler chicken samples.

*P. stuartii* isolates from broiler chickens	No. of Isolates	MAR index
AMP25, AMC30, AT30, C30, CIP5, NX10, CTX30, CX30, K30, TE30, IMP10, AZM 30, E15, CL10, PB300, COT25	1	1.0
AMP25, AMC30, C30, NX10, CTX30, CX30, GEN10, K30, TE30, IMP10, AZM30, E15, CL10, PB300, COT25	1	0.88
AMP25, AMC30, C30, CIP5, CTX30, CX30, GEN10, K30, TE30, IMP10, AZM30, E15, CL10, PB300, COT25	1	0.88
AMP25, AMC30, C30, CIP5, NX10, CTX30, CX30, K30, TE30, IMP10, AZM30, E 15, CL10, PB300, COT25	1	0.88
AMP25, AMC30, AT30, C30, CTX, CX30, K30, TE30, IMP10, AZM30, E15, CL10, PB300, COT25	1	0.82
AMP25, AMC30, C30, CTX, CX30, GEN10, K30, TE30, IMP10, AZM30, E15, CL 10, PB300, COT25	1	0.82
AMP25, AMC30, C30, CTX30, CX30, K30, TE30, IMP10, AZM30, E15, CL10, PB300, COT25	2	0.76
AMP25, C30, NX10, CX30, K30, TE30, IMP10, AZM30, E15, CL10, PB300, COT 25	1	0.71
AMP25, AMC30, C30, CX30, K30, TE30, AZM30, E15, CL10, PB300, COT25	1	0.64

In response to this concern, WGS was initiated to identify plasmids, ARGs, and virulence factors potentially linked to community-acquired infections. Two isolates (strain ps_nstu_001 and ps_nstu_002) were selected for WGS based on their high antibiotic resistance profiles. During the antibiotic susceptibility test, the *P. stuartii* ps_nstu_001 strain exhibited resistance to all 17 antibiotics tested, resulting in a MAR index of 1.00 and classifying it as a pan-drug-resistant (PDR) strain, as depicted in Supplementary Figure S1A. However, the *P. stuartii* ps_nstu_002 strain confers resistance to 13 antibiotics, intermediate to three, and is sensitive to only one antibiotic, Aztreonam, with a MAR index of 0.76, shown in Supplementary Figure S1B.

### Quality Validation of The Assembled Genome and Detailed N50 Analysis

The raw data obtained from WGS were mapped against the reference genome, which revealed 89.04% of the ps_nstu_001 genome and 91.51% of the ps_nstu_002 genome were covered by at least one read. After that, the raw reads were trimmed, and the output is presented in Supplementary Table S3, S4. Sequencing of ps_nstu_001 isolate generated 0.90 Gb of high-quality paired-end data, with a Q30 score of 94.9%, indicating excellent base-calling accuracy. Sequencing of isolate ps_nstu_002 generated 0.52 Gb of high-quality paired-end data with a Q30 score of 87.4%, indicating sufficient sequencing depth and acceptable base-calling accuracy for downstream analyses. The genome coverage, Q30 value, and raw data volume are given in Table 4. The trimmed paired data were then assembled into contig.fasta for subsequent annotation and characterization. Genome assembly quality for both the *P. stuartii* isolates was validated using BUSCO, showing a high level of completeness 99.8% (439/440 essential genes) with minimal gaps error. This result was presented in Supplementary Table S5. The assembled genome of *P. stuartii* ps_nstu_001 has a total length of 4,270,008 base pairs, while *P. stuartii* ps_nstu_002 had a slightly larger genome of 4,380,804 bp. Both genomes exhibited a GC content of 41.25% (Table 5). The N50 values: 209,619 bp for contig 9 and 143,501 bp for contig 8 indicate that half of each genome assembly is contained within contigs of at least these lengths. This suggested that a significant portion of the genome was captured within a single, large contig, which can facilitate downstream analyses and reduce assembly complexity (Ondov et al., 2016).

**Table 4:** Sequencing quality matrix and genome coverage of the isolates.

Isolate	Raw data volume (gb)	Q30 (%)	Coverage (%)
ps_nstu_001	0.90	94.9	89.04%
ps_nstu_002	0.52	87.4	91.51%

**Table 5:** Quality assessment of the assembled contig.fasta file from *Providencia stuartii*.

Assembly	*Providencia stuartii* ps_nstu_001	*Providencia stuartii* ps_nstu_002
# contigs (≥ 0 bp)	156	150
# contigs (≥ 1000 bp)	81	80
# contigs (≥ 5000 bp)	55	47
# contigs (≥ 10000 bp)	44	44
# contigs (≥ 25000 bp)	35	37
# contigs (≥ 50000 bp)	25	26
Total length (≥ 0 bp)	4270008	4380804
Total length (≥ 1000 bp)	4237986	4349585
Total length (≥ 5000 bp)	4177002	4271187
Total length (≥ 10000 bp)	4098572	4247492
Total length (≥ 25000 bp)	3954751	4133972
Total length (≥ 50000 bp)	3602175	3809910
# contigs	104	105
Largest contig	334169	556420
Total length	4254581	4366725
GC (%)	41.25	41.25
N50	209619	143501
N90	34717	31703
auN	174442.8	213016.7
L50	9	8
L90	31	30
# N's per 100 kbp	0.00	0

### Identity Validation and Mapping of Genetic Elements

The two studied isolates were identified as *P. stuartii* using KmerFinder, with further confirmation provided by Average Nucleotide Identity (ANI) and Basic Local Alignment Search Tool-Nucleotide (BLASTn), both revealing high similarity (>99%) with reference genomes. Identity and similarity of the isolates are presented in Table 6. This identification was additionally supported by *16S* rRNA sequencing, firmly establishing the isolate as *P. stuartii*. Following these confirmations, the isolates were designated as *P. stuartii* ps_nstu_001 strain and *P. stuartii* ps_nstu_002 strain.

**Table 6:** Identification of the test isolates.

(a) Average nucleotide identity (ANI)			
Isolate	Reference	ANI (%)	
*Providencia stuartii* ps_nstu_001	NZ_JADSTB010000001.1	99.3081	
	PGGX01000001.1	99.2896	
*Providencia stuartii* ps_nstu_002	JAVRQF010000001.1	99.8743	
	CAXOTU010000001.1	99.7105	

(b) BLASTn results			
Isolate	Matched isolate	Percent identity (%)	e value
*Providencia stuartii* ps_nstu_001	NZ_JADSTB010000003.1	99.575	0.00
	PGGX01000004.1	99.581	0.00
*Providencia stuartii* ps_nstu_002	JAVRQF010000037.1	100.00	0.00
	CAXOTU010000003.1	99.271	0.00

**Fig. 1:**
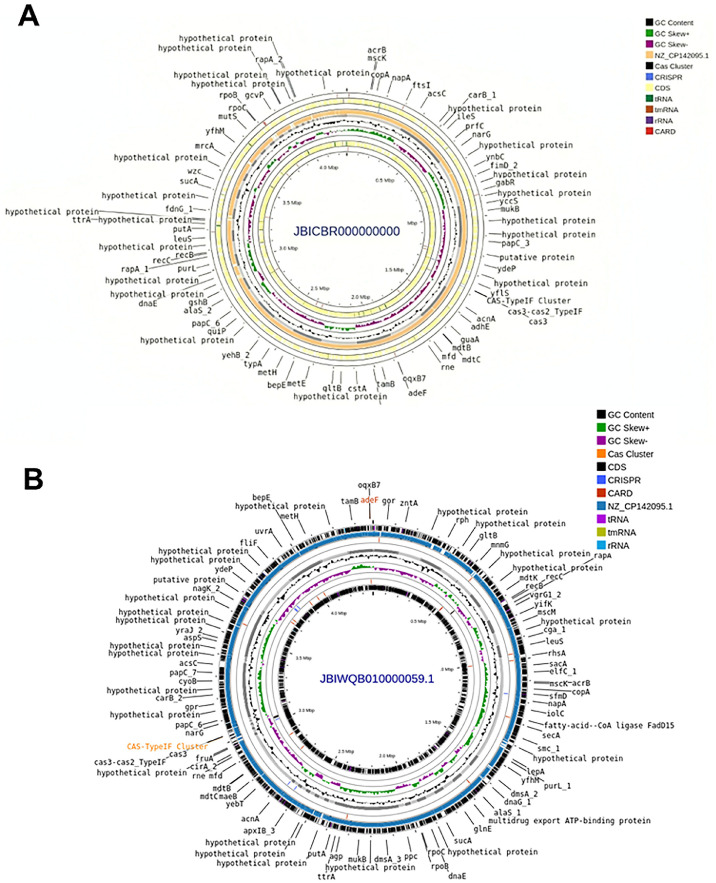
Circular annotation of *P. stuartii* ps_nstu_001 genome (JBICBR000000000) and *P. stuartii* ps_nstu_002 genome (JBIWQB000000000). (A) Here grey color is our study isolate and orange color depicted as reference genome (NZ_CP142095.1). Black color showing the annotated genes present in the genome. (B) Here grey color is our study isolate and blue color depicted as reference genome (NZ_CP142095.1). Black color showing the annotated genes present in the genome.

Draft genome analysis indicated high completeness (94.6%) and low contamination (2.32%) of *P. stuartii* ps_nstu_001, while *P. stuartii* ps_nstu_002 exhibited 96.37% completeness with 3.55% contamination, reflecting the genome's quality and consistency. Genomic features of *P. stuartii* ps_nstu_001 based on Rapid Annotations using Subsystems Technology toolkit (RASTtk) and rapid prokaryotic genome annotation (Prokka). Detailed genome annotation is reported in Supplementary Figure S2 and Table 7. In functional categorization via Rapid Annotations using Subsystem Technology (RAST), approximately 72% of the ps_nstu_001 genome was associated with defined biological processes, while 52% of the ps_nstu_002 genome was similarly annotated. Notably, 28% hypothetical proteins (HPs) were identified in *P. stuartii* ps_nstu_001 and 48% HPs were reported in *P. stuartii* ps_nstu_002, hinting at functional diversity, evolutionary adaptation, and the presence of novel genes or proteins of potential importance. Structural diversity and key genomic features of both isolates were illustrated in Fig. 1**.** The draft genome sequence of ps_nstu_001 and nstu_002 has been deposited in DDBJ/ENA/GenBank (NCBI) under accession numbers JBICBR000000000 and JBIWQB000000000, respectively.

**Table 7:** General features of the annotated genome.

RASTtk Annotation			Prokka Annotation		
ps_nstu_001		ps_nstu_002	ps_nstu_001		ps_nstu_002
Genome	*Providencia stuartii* ps_nstu _001 (Taxonomy ID: 588)	*Providencia stuartii* ps_ nstu_002 (Taxonomy ID: 588)	contigs	156	150
Size	4,270,008	4,380,804	bases	4270008	4380804
GC Content	41.2	41.3	CDS	3906	4001
N50	209619	185444	CRISPR	4	N/A
L50	9	7	rRNA	2	5
Contigs	156	150	tRNA	54	53
Subsystems	335	336	tmRNA	1	1
CDS	4265	4377	Repeat_region	N/A	6
RNAs	60	59

### Investigation of Anti-microbial Resistant Genes and Associated Mobile Genetic Elements

**Fig. 2:**
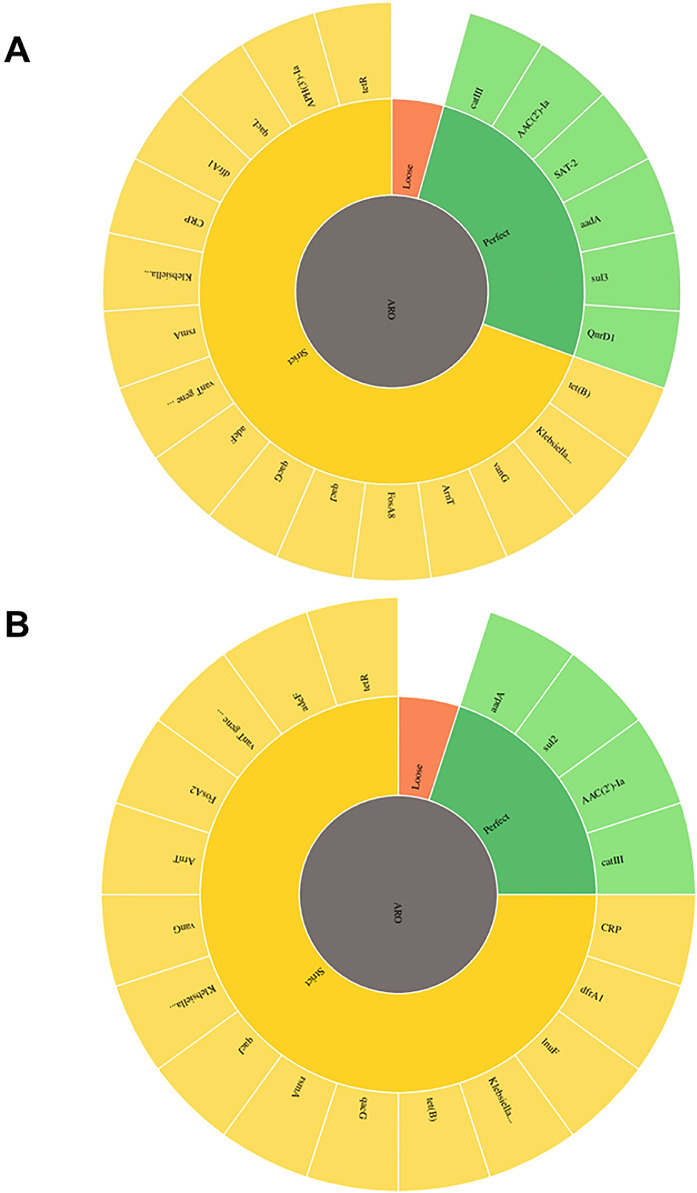
Antibiotic Resistance Determinants in (A) *P. stuartii* ps_nstu_001 (B) *P. stuartii* ps_nstu_002 isolates in terms of CARD database categorized based on confidence levels. The different colors indicate the strength of the match: green (perfect), yellow (strict), and red (loose).

**Fig. 3:**
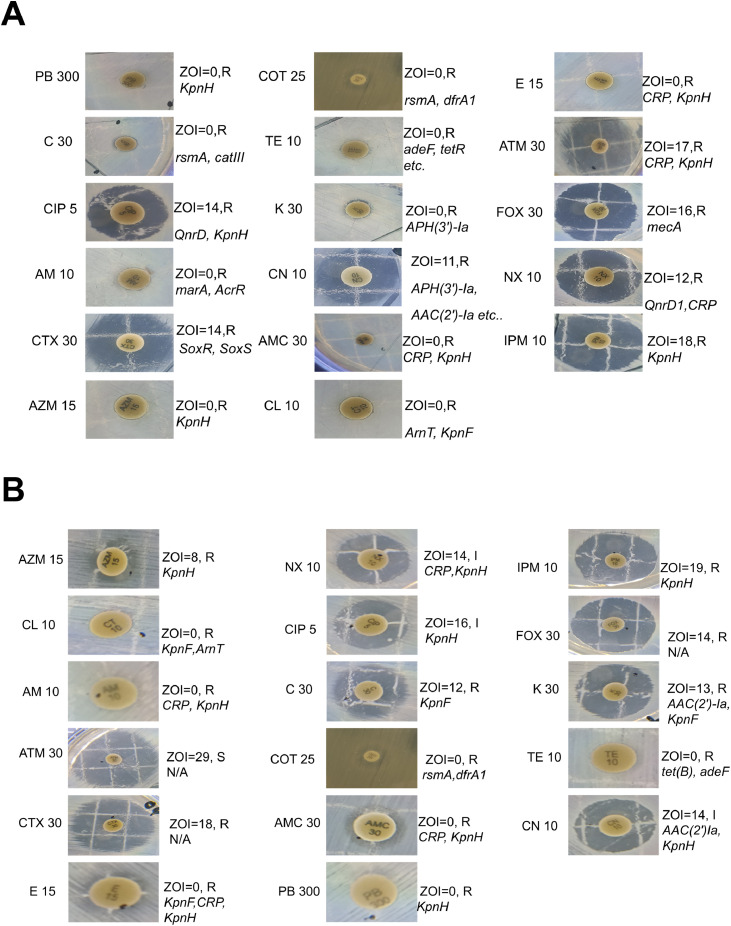
Correlation of Antimicrobial Resistance (AMR) Patterns with Genotypic Determinants in *P. stuartii* isolates. Antimicrobial disk diffusion test (zone of inhibition) is labeled with the corresponding antibiotic and associated resistance genes. The resistance genes identified for each antibiotic are indicated next to their respective images.

The two *P. stuartii* strains were found to harbor a diverse array of antibiotic resistance genes, each contributing to their MDR phenotype, as presented in Fig. 2. The identified antibiotic resistance genes, their associated drug classes, and the corresponding target antibiotics were analyzed using the CARD summarized in Table 8. Both strains encode aminoglycoside resistance genes, including *aac(2′)Ia, aadA, aph(3′)Ia, aadA36*, and *aadA1*. Additionally, fluoroquinolone resistance was conferred by different sets of genes: *adeF, rsmA, crp*, and q*nrD1* were detected in ps_nstu_001, while *aadA* was identified in ps_nstu_002. The genes *kpnF, kpnH* from *kpnEF* efflux pump are also observed in both strains. Notably, each strain also possesses unique resistance determinants. The nucleoside resistance gene *sat-2* was exclusively identified in ps_nstu_001, whereas the lincosamide resistance gene *lnuF* was found only in ps_nstu_002. While the two strains share similar resistance gene categories (e.g., sulfonamide, phosphonic acid), the specific variants differ: *sul3* and *fosA8* in ps_nstu_001 versus *sul2* and *fosA2* in ps_nstu_002. To correlate phenotypic antibiotic susceptibility patterns with genotypic determinants, a comparative visualization for both *P. stuartii* isolates is illustrated in Fig. 3**.**

**Table 8:** Antibiotic resistance genes, drug class, and respective antibiotics according to CARD analysis.

*Providencia stuartii* ps_nstu_001			*Providencia stuartii* ps_nstu_002		
Class	Gene	Antimicrobial Resistance	Class	Gene	Antimicrobial Resistance
Aminoglycoside	*AAC (2′)-Ia*	dibekacin; netilmicin; tobramycin; 6′-N-ethylnetilmicin; gentamicin	Aminoglycoside	*aadA*	spectinomycin; streptomycin
	*APH (3′)-Ia*	neomycin; ribostamycin; kanamycin A; gentamicin B; paromomycin; lividomycin; gentamicin		*AAC (2′)-Ia*	dibekacin; netilmicin; tobramycin; 6′-N-ethylnetilmicin; gentamicin
Nucleoside	*SAT-2*	streptothricin	Lincosamide	*lnuF*	lincomycin; clindamycin
Phenicol	*catIII*	azidamfenicol; chloramphenicol; thiamphenicol	Phenicol	*catIII*	azidamfenicol; chloramphenicol; thiamphenicol
Sulfonamide	*sul3*	sulfadiazine; sulfadimidine; sulfadoxine; sulfamethoxazole; sulfisoxazole; sulfacetamide; mafenide; sulfasalazine; sulfamethizole	Sulfonamide	*Sul2*	sulfadiazine; sulfadimidine; sulfadoxine; sulfamethoxazole; sulfisoxazole; sulfacetamide; mafenide; sulfasalazine; sulfamethizole
Fluoroquinolone	*QnrD1*	ciprofloxacin; levofloxacin; moxifloxacin; gatifloxacin; nalidixic acid; norfloxacin; sparfloxacin	Fluoroquinolone	aadA	spectinomycin; streptomycin
	*rsmA*	trimethoprim; chloramphenicol		*rsmA*	trimethoprim; chloramphenicol
Tetracycline	*tet(B), tet(R), adeF*	tetracycline	Tetracycline	*tet(B), tet(R), adeF, KpnF*	Tetracycline
Macrolide	*KpnF*	erythromycin; streptomycin; tetracycline; cefepime; ceftriaxone; rifampin; colistin A; colistin B; triclosan; benzalkonium chloride; chlorhexidine	Macrolide	*KpnF, KpnH*	tetracycline; cefepime; ceftriaxone; rifampin; colistin A; colistin B; erythromycin; gentamicin C; ciprofloxacin; spectinomycin; streptomycin; tobramycin; ceftazidime; azithromycin;
	*CRP*	erythromycin; cloxacillin; oxacillin; norfloxacin		*CRP*	erythromycin; cloxacillin; oxacillin; norfloxacin
Cephalosporin	*Klebsiella pneumoniae KpnF, KpnH*	Cefepime, Ceftriaxone, Ceftazidime	Cephalosporin	*Klebsiella pneumoniae KpnF, KpnH*	Cefepime, Ceftriaxone, Ceftazidime
Peptide	*ArnT*	colistin A; colistin B	Peptide	*ArnT*	colistin A; colistin B
Rifamycin	*Klebsiella pneumoniae KpnF*	rifampin	Rifamycin	*Klebsiella pneumoniae KpnF*	rifampin
Disinfectant	*Klebsiella pneumoniae KpnF, qacG, qacL, qacJ*	ciprofloxacin; levofloxacin; moxifloxacin; gatifloxacin; nalidixic acid; norfloxacin; sparfloxacin, Triclosan, Benzalkonium chloride, Chlorhexidine	Disinfectant	*Klebsiella pneumoniae KpnF, qacG, qacJ*	ciprofloxacin; levofloxacin; moxifloxacin; gatifloxacin; nalidixic acid; norfloxacin; sparfloxacin, Triclosan, Benzalkonium chloride, Chlorhexidine
Glycopeptide	*vanG, vanT gene in vanG cluster*	vancomycin	Glycopeptide	*vanG, vanT gene in vanG cluster*	vancomycin
Phosphonic acid	*FosA8*	fosfomycin	Phosphonic acid	*FosA2*	fosfomycin
Diaminopyrimidine	*dfrA1*	trimethoprim	Diaminopyrimidine	*dfrA1, rsmA*	Trimethoprim, chloramphenicol
Carbapenem	*Klebsiella pneumoniae KpnH*	erythromycin; ciprofloxacin; spectinomycin; streptomycin; tobramycin; ertapenem; azithromycin; imipenem; polymyxin B; norfloxacin; gentamicin	Carbapenem	*Klebsiella pneumoniae KpnH*	erythromycin; ciprofloxacin; spectinomycin; streptomycin; tobramycin; ertapenem; azithromycin; imipenem; polymyxin B; norfloxacin; gentamicin
Penicillin Beta lactam	*CRP*	erythromycin; cloxacillin; oxacillin; norfloxacin	Penicillin Beta lactam	*CRP, KpnH*	erythromycin; cloxacillin; oxacillin; norfloxacin

Genomic analysis of ps_nstu_001 revealed the presence of three MGEs, shown in Table 9 ISEc46, ISYal1; as insertion sequence known for facilitating gene mobility, and Tn4352, a composite transposon often associated with the horizontal transfer of antibiotic resistance genes, whereas two insertion sequences (ISYal1, ISEhe4) was detected in strain ps_nstu_002. In the meantime, AB434 plasmid (96.7% identity) was detected in strain ps_nstu_001, while the ps_nstu_002 strain harbors the Col3M plasmid (93.63% identity) and Col pHAD28 plasmid (90.076% identity), a small, non-conjugative element that may carry AMR genes.

**Table 9:** Distribution and Characterization of Mobile Genetic Elements and Plasmids in *P. stuartii* ps_nstu_001 and ps_nstu_002 isolates.

Isolate	Name	Type
ps_nstu_001	ISEc46	Insertion sequence
	ISYal1	Insertion sequence
	Tn4352	Composite transposon
	AB434	Plasmid
ps_nstu_002	ISYal1	Insertion sequence
	ISEhe4	Insertion sequence
	Col3M	Plasmid
	Col pHAD28	Plasmid

### Analysis of Metal Resistance Determinants and Multi-Locus Sequence Typing

Both *P. stuartii* strains were found to harbor genes conferring resistance to both metals and biocides as *terD and qacL genes*. The MLST analysis showed that *P. stuartii* strain ps_nstu_001 revealed the closest sequence type ST 80 with the following allele numbers for the analyzed loci: *fusA_7, gyrB_15*, and *lepA_19*. Sixty percent of loci (3/5) matched with this ST 80. For *P. stuartii* strain ps_nstu_002, MLST analysis identified alleles for key housekeeping genes: *fusA (7), gyrB (18), ileS (19), lepA (18)*, and *leuS (18*). The strain showed the highest similarity to sequence type ST 18, with 80% loci identity, suggesting potential evolutionary divergence or novel sequence variation.

### The Pangenome Analysis of *P. Stuartii* Isolates

**Fig. 4:**
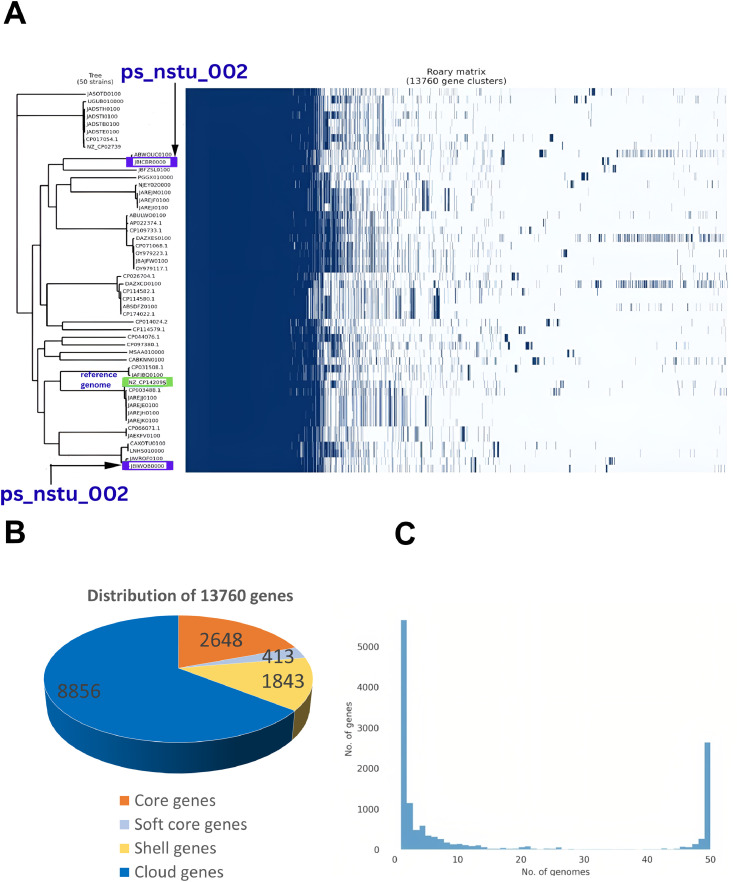
(A) Pangenome roary matrix analysis and phylogenetic tree of 50 *P. stuartii* isolates. Blue colored 2 samples (JBICBR000000000, JBIWQB000000000) are our study sample from poultry and other 48 samples are from clinical isolates. Green colored isolate is reference genome (NZ_CP142095.1). (B) Pie chart of pangenomic *P. stuartii* visually showing the distribution of genes into different categories based on their presence across the 50 genomes. (C) Histogram analysis distribution of gene frequencies across the 50 *P. stuartii* genomes. Core genes appear in most genomes (left peak), unique genes in single genomes (right peak), and accessory genes show variable presence (middle region).

**Fig. 5:**
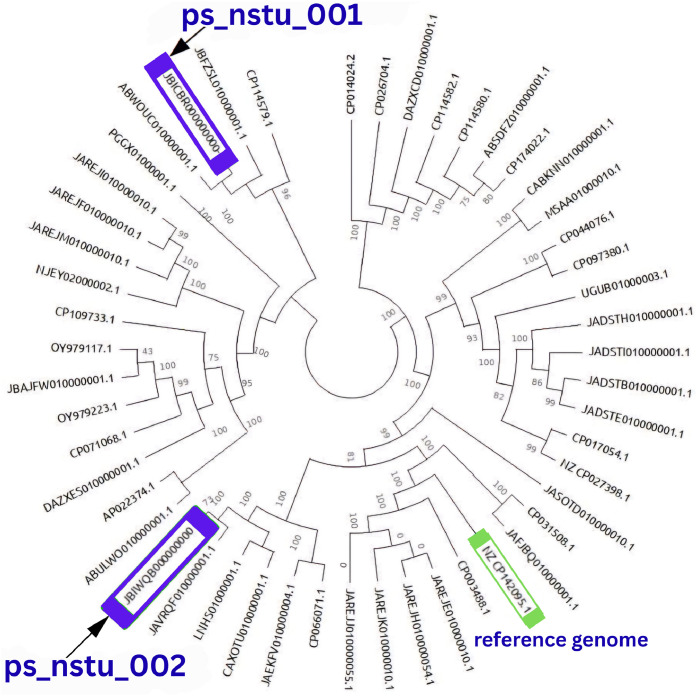
SNP- based phylogenetic relationship among 50 isolates of *P. stuartii*. Blue and Green labeled isolates are the studied isolates (JBICBR000000000, JBIWQB000000000) and reference genome (NZ_CP142095.1) respectively.

An open pangenome was reported for *P. stuartii* based on pangenome analysis, indicating that new genes continue to be acquired over time. A small core genome, consisting of 2,648 genes among 13,760 genes, was observed. Consequently, 413 soft core genes, 1,843 shell genes, and 8,856 cloud genes were identified, which are illustrated in Fig. 4**B,** 4**C.** Pangenome-based phylogenetic analysis is depicted in Fig. 4**A**, revealing that ps_nstu_001 (JBICBR000000000) clustered within a distinct clade with *P. stuartii* strain ABWOUC01 (ABWOUC010000001.1), which was isolated from a human urine sample in the USA and is distantly related to *P. stuartii* strain fly-386 (JBFZSL010000001.1) from flies in China. This suggests that ps_nstu_001 is phylogenetically proximate to a human clinical isolate, whereas it has diverged from other strains associated with humans and insects. However, the second study isolate, *P. stuartii* strain ps_nstu_002 (JBIWQB000000000), isolated from a broiler stool sample in Noakhali, Bangladesh, was reported to cluster closely with *P. stuartii* strain NIB002 (JAVRQF010000001.1), which was isolated from a diarrheal patient in Bangladesh. Furthermore, a distant phylogenetic relationship with several other human-derived isolates, including *P. stuartii* FDAARGOS_1040 and FDAARGOS_1035 from clinical samples in the USA, *P. stuartii* strain 50655837 (LNHS01000001.1) from a urine sample in Norway, and *P. stuartii* strain uvzsr-PT_24_00014-A12-AR_24_00000940 (CAXOTU010000001.1) from a diseased patient in Slovakia. In addition, the SNP-based phylogenetic relationship, shown in Fig. 5**,** also reassured the relationship patterns among the isolates, where ps_nstu_001 creates a clade with *P. stuartii* strain ABWOUC01 and ps_nstu_002 is closely linked with *P. stuartii* strain NIB002.

**Fig. 6:**
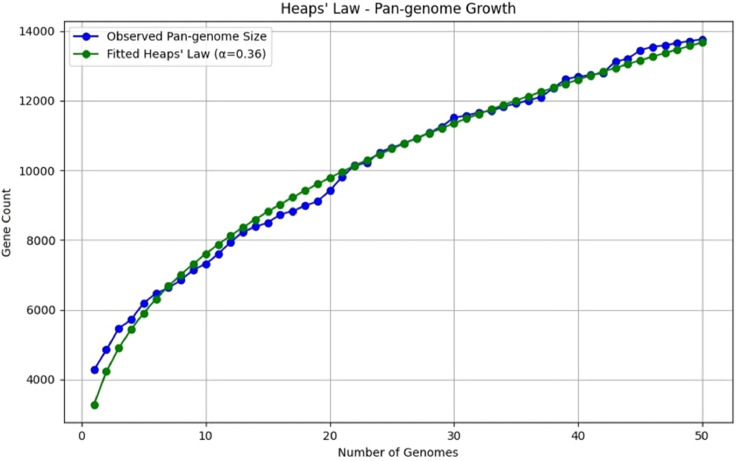
Heap's Law plot showing the growth of a pangenome of 50 *P. stuartii* isolates. Blue line depicted as observed mean pangenome size and green line represented as Fitted heaps law.

The pan-genome growth analysis of *P. stuartii* follows Heaps’ Law, demonstrating how genetic diversity expands with the inclusion of additional genomes. The plot is illustrated in Fig. 6**.** The blue line depicts the observed pan-genome size, reflecting the accumulation of unique genes, whereas the green line represents the fitted Heaps’ Law model with an exponent α = 0.36.

### Genome-wide pathogenicity profiling and proteome comparison

**Fig. 7:**
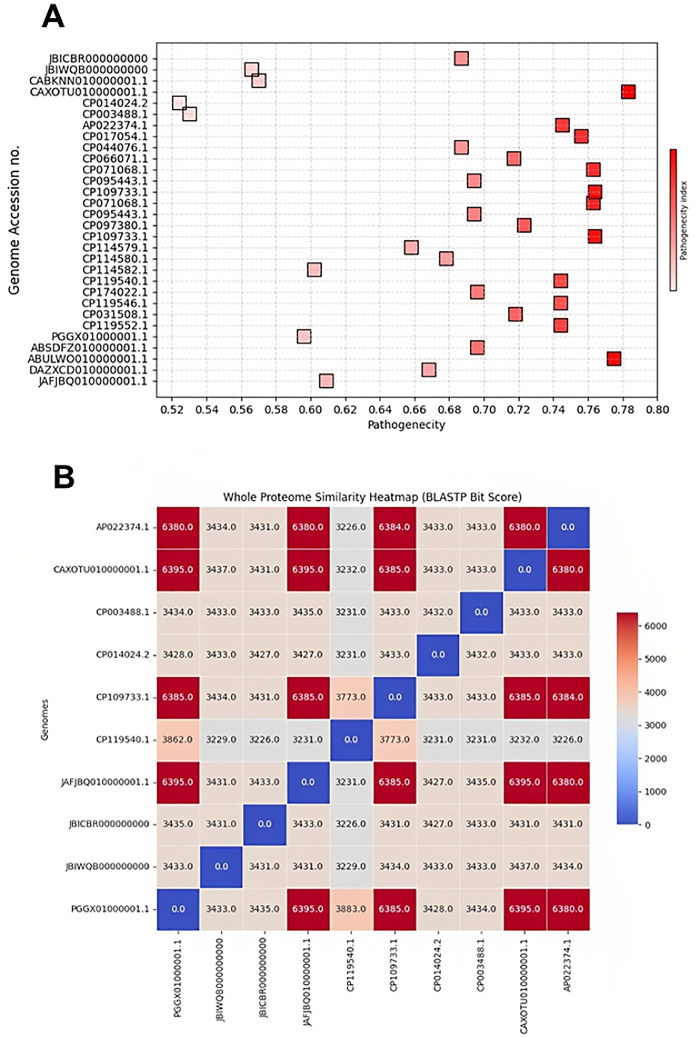
(A) Pathogenicity analysis of 30 representative *P. stuartii* isolates from diverse countries and sources, including the study isolates from this study. The x-axis represents the pathogenicity index, while the y-axis lists genome accession numbers. Each square represents a genome, with color intensity corresponding to pathogenicity levels- darker red indicates a higher pathogenicity index. (B) Comparative whole-proteome analysis of pathogenic and non-pathogenic *P. stuartii* isolates from various sources measured using BLASTP bit scores. The x-axis and y-axis denote genome accession numbers. Each cell indicates the similarity score between two genomes, with higher scores (red) representing greater proteomic similarity and lower scores (blue) indicating less similarity. Diagonal values (0.0) correspond to self-comparisons.

The pathogenicity profiling indicated CAXOTU010000001.1 as the most pathogenic strain and predicted to be a probable human pathogen with a probability of 0.783. Notably, all isolates were classified as human pathogens except five strains (accession no. PGGX01000001.1, CP003488.1, CP014024.2, CABKNN010000001.1, JBIWQB000000000), which originated from both human and non-human sources. The *P. stuartii* strain ps_nstu_001 exhibits a moderate to high probability (0.687) of being a human pathogen, supported by the enrichment of pathogenic protein families. In contrast, strain ps_nstu_002 exhibited a lower probability (0.566), indicating to be a probable non-human pathogen, but it is in the borderline of being human pathogen. The worldwide pathogenicity profiling of *P. Stuartii* species is shown in Fig. 7A, and the data are given in Supplementary Table S7. However, most of the strains, including ps_nstu_001 (JBICBR000000000), clustered in between 0.66 and 0.78. ps_nstu_002 (JBIWQB000000000) therefore represents a clear outline on the lower end of the pathogenicity scale. Interestingly, two human-derived isolates from Slovakia and the U.S.A. exhibited a high pathogenic profile and shared phylogenetic links with studied isolates (ABULWO010000001.1 with the strain ps_nstu_001 JBICBR000000000 and CAXOTU010000001.1 with ps_nstu_002, JBIWQB000000000).

Furthermore, strains from non-human sources displayed a similar proteomic composition, distinct from those isolated from human sources which is illustrated in Fig. 7**B**. Additionally, ps_nstu_001 JBICBR000000000 showed highest proteomic similarity with PGGX01000001.1 and ps_nstu_002 JBIWQB000000000 showed the highest proteomic similarity with CAXOTU010000001.1 in bidirectional BLASTp among selected ten human and non-human pathogenic isolates indicate the ability to move between host species and the values are given in Supplementary Table S8.

## Discussion

*Providencia spp.* cause human and animal gastroenteritis outbreaks, supported by epidemiologic and molecular evidence. Also, MDR and PDR in this genus is rising worldwide. As a result, significant diagnostic complexities and therapeutic challenges are increasing globally today (Janda et al., 2025). Additionally, *P. stuartii* is continuously involved in nosocomial infections, particularly in intensive care and burn units, where it causes infections with high mortality and morbidity rates (Liu et al., 2020). Multiple major antibiotic classes, such as aminoglycosides, penicillins, and polymyxins, which frequently produce extended-spectrum beta-lactamases (ESBLs), are increasingly resistant to *P. stuartii*, contributing to its critical resistance profile and complicating therapeutic strategies. Although it has clinical significance, the ecology of *P. stuartii* spreads across hospitals and into surrounding natural reservoirs, such as soil, water, insects, and diverse animal hosts, including poultry, which may serve as reservoirs and vectors for resistant strains (Abdallah and Balshi, 2018). Moreover, analyzing the zoonotic transmission dynamics through comprehensive genomic studies, such as pangenomic diversity and AMR mechanisms of poultry-derived *P. stuartii,* remains limited, particularly in Noakhali, Bangladesh, where intensive antibiotics are used in livestock.

In this study, we have chosen small-scale farms from the suburban area. All of those farms supply chickens to the wholesale market in Noakhali. Additionally, these farms are operated by local stakeholders, where veterans are rarely involved in diagnosed diseases or prescribed medicine. However, *P. stuartii* isolates from broiler samples in Noakhali, Bangladesh, showed alarming MDR and PDR, with nine of ten isolates displaying MAR indices above 0.7. Particularly, strain ps_nstu_001 showed resistance to all 17 tested antibiotics, while ps_nstu_002 was resistant to 13, intermediate to 3, and sensitive to 1 antibiotic only. This scenario highlights the crucial intersection of resistance mechanisms in poultry-associated strains. While observing the resistance profile, both strains were resistant to last-resort antibiotics such as carbapenems, polymyxins, which highlights the reduction in efficacy of current therapeutic options (Lu et al., 2023). The resistance of ps_nstu_001 and ps_nstu_002 to colistin and imipenem is concerning, as it is classified as a carbapenem-resistant Enterobacterales. The strain ps_nstu_002 is sensitive to aztreonam with a zone of inhibition measuring 29 mm, indicating susceptibility according to CLSI guidelines (susceptibility breakpoint ≥ 22 mm), suggesting that it inhibits its growth at standard clinical doses. It also indicates the absence of beta-lactamases, such as ESBLs, *bla*KPC, or *bla*AmpC, which can hydrolyze aztreonam (Xavier Sáez-Llorens, 2006).

According to WGS, the high coverage and sequencing quality observed for both *P. stuartii* isolates (ps_nstu_001 with 89% and 94%; ps_nstu_002 with 91% and 87%) enabled robust assembly and annotation, with completeness validated by BUSCO analyses at 99%. This score exceeds the 98.6% threshold identified by (Farooq and Rafique, 2025) for top decile pathogenic genomes and the 95% quality filter applied in recent *P. stuartii* population studies (Arcari et al., 2024). Furthermore, the high coverage breadth (87-94%) aligns with clinical validation standards for accurate variant calling and resistance gene detection in hospital-acquired pathogens (Wick et al., 2023). ANI and BLASTn analyses showed significant results, with the isolates showing up to 99% nucleotide similarity with clinically pathogenic isolates. According to BLASTn results, *P. stuartii* ps_nstu_002 was identical (100%) to *P. stuartii* strain NIB002 (JAVRQF010000001.1), previously isolated from a diarrheal patient in Bangladesh (https://www.ncbi.nlm.nih.gov/bioproject/PRJNA1017985/). This indicates that both isolates share identical or nearly identical genomic sequences and suggests that ps_nstu_002 may have a similar origin, virulence potential, or clinical relevance like strain NIB002. However, according to AMR Finder tool, both the isolates harbored a diverse array of resistance genes, including aminoglycosides (*aac(2′)-Ia, aadA*), fluoroquinolones (*qnrD1, adeF*), sulfonamides (*sul2, sul3*), and β-lactams (*KpnH, CRP*). The fluoroquinolone genes encode proteins that protect DNA gyrase and topoisomerase IV, enzymes essential for DNA replication, from fluoroquinolone antibiotics, allowing the bacteria to continue replicating in their presence (Piekarska, 2018). The *dfrA1* gene encodes dihydrofolate reductase, an enzyme that bypasses the inhibitory effect of trimethoprim-sulfamethoxazole on folic acid synthesis, enabling the bacteria to grow in the presence of these antibiotics (Ojo et al., 2002). The *KpnH* efflux pump plays a crucial role in the synthesis of the bacterial capsule and contributes to colistin resistance (Baron et al., 2016). Both strains were also found to contain the *catA3* gene, which encodes chloramphenicol acetyltransferase, an enzyme that inactivates chloramphenicol by acetylation and makes the antibiotic ineffective (Schwarz et al., 2004). Notably the two strains harbors carbapenem and penicillin beta-lactam genes (*CRP, KpnH*), which encode β-lactamase enzymes that hydrolyze these antibiotics and render them ineffective (Bush and Bradford, 2016). However, Carbapenems, such as *KpnH* are a subclass of beta-lactamases that degrade carbapenem antibiotics, making infections difficult to treat (Dossouvi and Ametepe, 2024). The ps_nstu_001 strain was found to carry MGEs (ISEc46, ISYal1, Tn4352), which are insertion sequences and composite transposons and ps_nstu_002 carry two insertion sequences (ISYal1, ISEhe4). Insertion sequences are short DNA segments capable of moving within the genome, whereas composite transposons are larger elements that carry additional genes, associated with antibiotic resistance or virulence (Khedkar et al., 2022). While, ps_nstu_001 found to carry AB434 plasmid; another strain ps_nstu_002 harbours the Col3M (carry quinolone resistance genes qnrD1 or qnrD3 in *Proteus* and *Escherichia* species) and Col pHAD28 (carry *aac(6′)-Ib* aminoglycoside resistance gene) plasmid suggesting about the ongoing horizontal gene transfer, facilitating the rapid transmission of resistance determinants (De Belder et al., 2023). These MGEs and plasmids are responsible for horizontal gene transfer. A recent study demonstrated that *E. coli* isolated from poultry also share a comparable phenotype with zoonotic transmission potential (Akter et al., 2026).

Additionally, the strain ps_nstu_001 carries a metal resistance gene (*terD*), which encodes a membrane protein involved in tellurium resistance (Mondol et al., 2024) and a biocide resistance gene (*qacL*), which encodes an efflux pump that expels quaternary ammonium compounds, commonly used as disinfectants. This type of gene indicates adaptive ability to diverse environmental stress, which may further support persistence and spread in agricultural settings (Mondol et al., 2024). Generally, *P. stuartii,* including various STs, is known to cause nosocomial infections, particularly urinary tract infections in hospital patients with long-term indwelling catheters (Zavascki et al., 2012). The MLST analyses showed that ps_nstu_001 is closely matched with ST 80, and ps_nstu_002 matched with ST 18. This sequence typing is essential for tracking the transmission and evolution of these bacteria, enabling better surveillance and control of bacterial infections (Le et al., 2020). No outbreaks associated with ST80 and ST18 were reported in previous studies.

The pangenome analyses revealed a small core genome with 2648 genes, which indicates continuous gene acquisition and substantial genetic diversity within the species (Rouli et al., 2015). The small core genome and large accessory genome indicate that *P. stuartii* is still acquiring new genes, leading to genetic diversity (Mondol et al., 2025). The SNP-based phylogenetic relationship of ps_nstu_001 to a clinically pathogenic isolate from a human urine sample in the U.S.A. and ps_nstu_002 to a Bangladeshi diarrheal patient isolate suggests potential zoonotic transmission and highlights the role of poultry as a reservoir for clinically relevant *P. stuartii* strains. Therefore, while ps_nstu_002 is not classified as a human pathogen at present, its genetic similarity to clinical isolates indicates a potential risk of acquiring virulence through horizontal gene transfer (Frost et al., 2005). Phylogenomic and evolutionary analyses suggest that poultry-derived *P. stuartii* isolates may have zoonotic potential. Although non-human isolates have diverged from human-associated strains, pangenome analysis revealed significant genetic variation, with a smaller core genome when non-human isolates were included. Overall, these findings suggest that while non-human *P. stuartii* strains currently exhibit lower pathogenic potential, their genetic proximity to human pathogens and ongoing genomic evolution may facilitate future zoonotic transmission (Price et al., 2012). Moreover, pangenome growth analysis reveals an open pangenome that follows Heaps law plot. The close alignment between observed and predicted values demonstrates the model’s reliability, with minor deviations attributable to specific strain-level differences or sampling biases (Tettelin et al., 2005). This open pangenome indicates that *P. stuartii* has a high capacity for genetic adaptation through the acquisition of foreign genes, which may contribute to increased pathogenicity and antibiotic resistance (Vernikos et al., 2015)**.** These findings align with a previous study indicating that bacterial species with open pan-genomes are often opportunistic pathogens with a strong environmental adaptability (Tettelin et al., 2005).

According to the pathogen finder tool, genome-wide pathogenicity profiling revealed notable differences in pathogenic potential among *P. stuartii* isolates from a poultry sample toward humans. The ps_nstu_001 strain exhibited a moderate probability of human pathogenicity (score: 0.687), followed by the presence of large pathogenic protein families, despite limited proteome coverage. Although the proteome coverage was low (∼0.77%), previous studies suggest that the presence of known virulence-associated protein families, even in small datasets, can be a strong indicator of pathogenic potential (Chen et al., 2016; Dobrindt et al., 2004). That means ps_nstu_001 possesses key virulence determinants associated with human infection. In contrast, ps_nstu_002 had a lower pathogenicity probability (0.566) and was classified as probable non-pathogenic to humans. Though it harbors various virulence-associated protein families and has a borderline pathogenicity score, it can be considered a probable human pathogen. Nevertheless, given its borderline probability score, ps_nstu_002 may acquire additional virulence factors through mechanisms such as horizontal gene transfer, making it an opportunistic pathogen, potentially elevating its pathogenic potential in the future (Frost et al., 2005).

Distinct pathogenicity patterns were revealed by comparative analysis across 30 *P. stuartii* genomes from diverse sources. In particular, some human isolates with high pathogenicity scores clustered phylogenetically with poultry-associated strains, indicating possible interspecies gene flow and zoonotic potential. Proteome comparisons further demonstrated that probable non-human isolates shared conserved protein profiles distinct from human-derived strains, whereas some poultry isolates showed high similarity to highly pathogenic human strains.

A recent study showed that, without using drugs or additives, poultry farming is profitable in small- and medium-scale poultry farms (Das et al., 2024). Thus, farmers should follow the method, as the adaptability and evolutionary plasticity of *P. stuartii* in this study revealed that poultry-associated strains may serve as reservoirs for virulence and resistance genes. The tendency of gene acquisition and the observed relevance in pathogenic protein families between human and non-human isolates highlight the risk of emergence of new, more virulent lineages with public health significance. Additionally, it may spread resistance genes, rendering other poultry pathogens untreatable. As a result, small-scale poultry farm owners suffer significant financial losses due to the growing prevalence of antibiotic-resistant pathogens. Lastly, continuous genomic surveillance is required to monitor the evolution of pathogenicity in *P. stuartii* populations across poultry environments.

## Conclusion

This study highlights the characterization of poultry-derived *P. stuartii* and WGS-based genomic analysis from small-scale poultry farms in Noakhali, Bangladesh. This reveals poultry as a reservoir of MDR *P. stuartii*, with the capacity for zoonotic transmission and for spreading antibiotic-resistant genes in poultry and humans. Consequently, it reveals key antimicrobial and metal resistance genes, along with other crucial elements involved in horizontal gene transfer. However, a small sample size and the inclusion of only small-scale poultry farms limit the broader applicability of these findings, as they don’t reflect the full picture of the poultry population in Bangladesh. Nevertheless, this study provided a firm foundation for the phenotypic and genomic surveillance of AMR in poultry across different types of poultry farms, enabling evaluation of AMR within a One Health framework and supporting the detection of emerging resistance genes and MDR pathogenic bacteria with zoonotic transmission capabilities. Lastly, this study supports strengthening public awareness, promoting prudent antimicrobial use in the poultry industry, and ensuring hygienic handling of poultry at live bird markets, all of which are essential to reducing AMR risks.

## Funding

The author extends the appreciation to the ‘Noakhali Science and Technology University-Research Cell’ Teachers’ grant of the budget year 2024-25 for funding this research work through the grant number (NSTU-RC-BGE-T-23-40 and NSTU-RC-BGE-T-23-41).

## Data availability

Sequenced data of the *16S* rRNA sequencing are available under the NCBI GenBank under the accession number PV239577, PV239578, PV239579, PV239580, PV239581, PV239582, and PV239583. The draft genome sequence of the isolate *P. stuartii* ps_nstu_001 and *P. stuartii* ps_nstu_002 has been submitted to DDBJ/ENA/GenBank of NCBI under the accession number JBICBR000000000 (https://www.ncbi.nlm.nih.gov/datasets/genome/GCF_043119525.1/) and JBIWQB000000000 (https://www.ncbi.nlm.nih.gov/datasets/genome/GCF_044751565.1/) and is publicly accessible. Additionally, the genomes utilized for secondary analysis were obtained from NCBI (https://www.ncbi.nlm.nih.gov/). *16* *s* rRNA sequence data are available in GenBank under the accession number PV239577, PV239578, PV239579, PV239580, PV239581, PV239582, and PV239583.

## Ethical permission

This study was approved by the Ethics and Research Review Committee of the Faculty of Science at Noakhali Science and Technology University (NSTU) under the reference number NSTU/SCI/EC/2023/186. A verbal consent was taken from the farm owners to collect bacterial sample from poultry chicken. All methods employed in this research were conducted in compliance with the applicable guidelines and regulations. Additionally, the present study is conducted in accordance with ARRIVE guidelines (https://arriveguidelines.org).

## CRediT authorship contribution statement

**Israt Jahan Asha:** Writing – review & editing, Writing – original draft, Methodology, Formal analysis, Data curation, Conceptualization. **Shipan Das Gupta:** Writing – review & editing, Writing – original draft, Visualization, Validation, Methodology, Investigation, Funding acquisition, Data curation, Conceptualization. **Md. Adnan Munim:** Writing – review & editing, Writing – original draft, Validation, Methodology. **Nurun Nahar Akter:** Writing – review & editing, Methodology, Formal analysis, Data curation. **Saheda Tamanna:** Writing – review & editing, Formal analysis. **Anisur Rahman:** Writing – review & editing, Formal analysis. **Al Imran:** Writing – review & editing, Formal analysis. **Shuvo Chandra Das:** Writing – review & editing, Conceptualization. **Md. Murad Hossain:** Writing – review & editing. **Mohammed Mafizul Islam:** Writing – review & editing. **Dhirendra Nath Barman:** Writing – review & editing, Writing – original draft, Visualization, Validation, Supervision, Resources, Project administration, Investigation, Funding acquisition, Data curation, Conceptualization.

## Disclosures

The authors declare no competing interests.

## Appendix Supplementary materials

Image, application 1

List of Supplementary Figures Supplementary Figure S1: Antibiogram test of (A) *P. stuartii* ps_nstu_001 and (B) *P. stuartii* ps_nstu_002 by Kirby-Bauer disk diffusion method using 17 antibiotics. (Ampicillin (AMP 25), Amoxicillin-clavulanic acid (AMC 30), Cefotaxime (CTX 30), Cefoxitin (CX 30), Ciprofloxacin (CIP 5), Norfloxacin (NX 10), Aztreonam (ATM 30), Gentamicin (GEN 10) and Kanamycin (K 30), Azithromycin (AZM 30), Erythromycin (E 10), Imipenem (IMP 10), Chloramphenicol (C 30), sulfonamides-Trimethoprim-Sulfamethoxazole (COT 25), Tetracycline (TE 30), Colistin (CL 10), and Polymyxin B (PB 300), (ZI= Zone of Inhibition in mm, *S*= sensitive, *R*= resistant, Zone of inhibition was interpreted according to CLSI, 2018). Image, image 2 dummy alt text

Supplementary Figure S2: The subsystem annotation of (A) *P. stuartii* ps_nstu_001 genome and (B) *P. stuartii* ps_nstu_002 genome. Image, image 3 dummy alt text

## Footnotes

Supplementary material associated with this article can be found, in the online version, at doi:10.1016/j.psj.2026.106602.
