# Supplementary material for: Emerging zoonotic risks: whole-genome sequencing reveals antimicrobial resistance and genomic diversity in Providencia stuartii isolated from broiler chickens in Noakhali, Bangladesh
Source: Poult Sci. 2026 Feb 10;105(5):106602. doi: 10.1016/j.psj.2026.106602 (PMC12925552; doi:10.1016/j.psj.2026.106602)
Supplement: Supplementary file 1 [file mmc1.docx]

Supplementary Table S1: Characteristics of poultry chickens.

| **Features** | **Sample 1 Broiler** | **Sample 2 Broiler** | **Sample 3 Broiler** | **Sample 4 Broiler** | **Sample 5 Broiler** | **Sample 6 Broiler** |
| --- | --- | --- | --- | --- | --- | --- |
| Age | 40 days | 280 days | 280 days | 30 days | 30 days | 300 days |
| Weight | 1.55 kg | 1.73 kg | 1.9 kg | 1.28 kg | 1.46 kg | 1.65 kg |
| Food supplied | Commercial poultry feed | Commercial poultry feed | Commercial poultry feed | Commercial poultry feed | Commercial poultry feed | Commercial poultry feed |
| Drinking water supplied | Deep tube well water | Deep tube well water | Deep tube well water | Deep tube well water | Deep tube well water | Deep tube well water |
| Health condition | Healthy | Healthy | Healthy | Healthy | Healthy | Healthy |
| Source of the chicken | Commercial poultry hatchery | Commercial poultry hatchery | Commercial poultry hatchery | Commercial poultry hatchery | Commercial poultry hatchery | Commercial poultry hatchery |

Supplementary Table S2: List of antibiotics and their classes used in the antibiotic susceptibility test.

| **Antibiotic classes** | **Antibiotics** |
| --- | --- |
| Penicillin | Ampicillin (AMP25) |
| β-lactam combination agents | Amoxicillin-clavulanic acid (AMC 30) |
| Cephalosporins | Cefotaxime (CTX30) (3rd generation)  Cefoxitin (CX30) (3rd generation) |
| Quinolones And Fluoroquinolones | Ciprofloxacin (CIP 5)  Norfloxacin (NX10) |
| Monobactams | Aztreonam (AT 30) |
| Aminoglycosides | Gentamicin (GEN 10)  Kanamycin (K 30) |
| Macrolides | Azithromycin (AZM 30)  Erythromycin (E 10) |
| Carbapenems | Imipenem (IMP 10) |
| Phenicol | Chloramphenicol (C30) |
| Folate pathway antagonists | Trimethoprim-Sulfamethoxazole (COT) |
| Tetracyclines | Tetracyclines (TE 30) |
| Polymyxin | Colistin (CL10)  Polymyxin B (PB 300) |

Supplementary Table S3: Fastqc report of *P. stuartii* ps_nstu_001 trimmed paired forward and reverse reads.

| **Measure** | **Output** | **Output** |
| --- | --- | --- |
| Filename | NSTU_PS_R1_TrmP.fastq.gz | NSTU_PS_R2_TrmP.fastq.gz |
| File type | Conventional base calls | Conventional base calls |
| Encoding | Sanger / Illumina 1.9 | Sanger / Illumina 1.9 |
| Total Sequences | 4724619 | 4724619 |
| Sequences flagged as poor quality | 0 | 0 |
| Sequence length | 35-151 | 35-151 |
| %GC | 41 | 41 |

Supplementary Table S4: Fastqc report of *P. stuartii* ps_nstu_002 trimmed paired forward and reverse reads.

| **Measure** | **Output** | **Output** |
| --- | --- | --- |
| Filename | trimmed_paired_R1.fastq.gz | trimmed_paired_R2.fastq.gz |
| File type | Conventional base calls | Conventional base calls |
| Encoding | Sanger / Illumina 1.9 | Sanger / Illumina 1.9 |
| Total Sequences | 1761645 | 1761645 |
| Sequences flagged as poor quality | 0 | 0 |
| Sequence length | 36-146 | 36-146 |
| %GC | 41 | 41 |

Supplementary Table S5: Assembly validation by BUSCO.

| **Metric** | ***Providencia stuartii* ps_nstu_001** | ***Providencia stuartii* ps_nstu_002** |
| --- | --- | --- |
| C (Complete BUSCOs) | 99.8% | 99.8% |
| S (Single-copy BUSCOs) | 99.1% | 98.9% |
| D (Duplicated BUSCOs) | 0.7% | 0.9% |
| F (Fragmented BUSCOs) | 0.0% | 0.0% |
| M (Missing BUSCOs) | 0.2% | 0.2% |
| n (Total BUSCO genes searched) | 440 | 440 |

Supplementary Table S6: Global Genome Samples for Pangenome Analysis of *P. stuartii*.

| **Category** | **Strain IDs** |
| --- | --- |
| Poultry Isolates | JBICBR000000000, JBIWQB000000000 |
| Clinical Isolates (Carbapenemase-producing) | NZ_CP142095.1 (blaNDM on IncC plasmid), OY979223.1 (Bulgaria), OY979117.1 (Bulgaria), CP026704.1 (Carbapenem Breakpoint project), CP109733.1 (CDC HAI-Seq), CP114580.1, CP071068.1 (MDR, ICU patient), CP174022.1 (CDC HAI-Seq), AP022374.1 (IMP-type MBL producing) |
| Clinical Isolates (General) | NZ_CP027398.1, CP114582.1, CP097380.1 (Microbe sample), CP017054.1 (urease-positive MDR), CP114579.1, CP044076.1, CP014024.2 (FDA-ARGOS), CP066071.1, CP031508.1, NJEY02000002.1, CP003488.1 (MRSN 2154), MSAA01000010.1, JAVRQF010000001.1, CABKNN010000001.1 (Human gut genome), CAXOTU010000001.1 (Slovak AMR surveillance), LNHS01000001.1 (Producing Enterobacteriaceae, Norway) |
| Clinical Isolates (Additional) | NZ_CP027362.1, NZ_CP027355.1, NZ_CP139846.1 (Microbe sample), NZ_CP156706.1, NZ_CP156662.1, NZ_CP029692.1 (Strain: SD134209), NZ_CP157170.1, NZ_CP048920.1 (Strain: SCU-101), NZ_CP051849.1, NZ_CP146670.1 (MS 85-1), ABWOUC010000001.1 (Urine, human, USA), DAZXCD010000001.1 (NDM-1-producing, Romania), ABULWO010000001.1 (Microbe sample, rectal swab, human, USA), ABSDFZ010000001.1 (Urine, human) |
| Research and Surveillance Projects | FDA-ARGOS: JAEKFV010000004.1, CP014024.2  CDC HAI-Seq: CP109733.1, CP174022.1  EDS-HAT: JADSTI010000001.1, JADSTE010000001.1, JADSTH010000001.1, JADSTB010000001.1, JAFJBQ010000001.1 |
| Other Sources | NCTC_3000: UGUB01000003.1  MRSN 2154: CP003488.1  Slovak AMR surveillance: CAXOTU010000001.1  Producing Enterobacteriaceae (Norway): LNHS01000001.1 |
| NDM-producing Providencia thailandensis (Ukraine) | AREJE010000010.1, JAREJJ010000055.1, JAREJH010000054.1, JAREJK010000010.1, JAREJM010000010.1, JAREJI010000010.1 |
| KPC-producing Enterics | PGGX01000001.1 |
| Genomic Catalog of Human Bladder Bacteria | JASOTD010000010.1 |
| Genomic Diversity of Carbapenemase-producing *Morganella morganii* | JBAJFW010000001.1 |
| NDM-1-producing Providencia stuartii (Romania) | DAZXES010000001.1, DAZXCD010000001.1 |

Supplementary Table S7: Pathogenicity profile of 30 *P. stuartii* isolates.

| **Genome Accession no.** | **Pathogenicity** |
| --- | --- |
| JAFJBQ010000001.1 | 0.609 |
| DAZXCD010000001.1 | 0.668 |
| ABULWO010000001.1 | 0.775 |
| ABSDFZ010000001.1 | 0.696 |
| PGGX01000001.1 | 0.596 |
| CP119552.1 | 0.744 |
| CP031508.1 | 0.718 |
| CP119546.1 | 0.744 |
| CP174022.1 | 0.696 |
| CP119540.1 | 0.744 |
| CP114582.1 | 0.602 |
| CP114580.1 | 0.678 |
| CP114579.1 | 0.658 |
| CP109733.1 | 0.764 |
| CP097380.1 | 0.723 |
| CP095443.1 | 0.694 |
| CP071068.1 | 0.763 |
| CP109733.1 | 0.764 |
| CP095443.1 | 0.694 |
| CP071068.1 | 0.763 |
| CP066071.1 | 0.717 |
| CP044076.1 | 0.687 |
| CP017054.1 | 0.756 |
| AP022374.1 | 0.745 |
| CP003488.1 | 0.53 |
| CP014024.2 | 0.524 |
| CAXOTU010000001.1 | 0.783 |
| CABKNN010000001.1 | 0.57 |
| JBIWQB000000000 | 0.566 |
| JBICBR000000000 | 0.687 |

Supplementary Table S8: Screening of 10 human and non-human pathogens for bidirectional Blastp.

| **Genome Accession no.** | **Probability of Human Pathogen** | **Prediction** |
| --- | --- | --- |
| JAFJBQ010000001.1 | 0.609 | Human pathogen |
| PGGX01000001.1 | 0.596 | Non-human pathogen |
| CP119540.1 | 0.744 | Human pathogen |
| CP109733.1 | 0.764 | Human pathogen |
| AP022374.1 | 0.745 | Human pathogen |
| CP003488.1 | 0.53 | Non-human pathogen |
| CP014024.2 | 0.524 | Non-human pathogen |
| CAXOTU010000001.1 | 0.783 | Human pathogen |
| JBIWQB000000000 | 0.566 | Non-human pathogen |
| JBICBR000000000 | 0.687 | Human pathogen |
