# Supplementary figures and images for: Emerging zoonotic risks: whole-genome sequencing reveals antimicrobial resistance and genomic diversity in Providencia stuartii isolated from broiler chickens in Noakhali, Bangladesh
Source: Poult Sci. 2026 Feb 10;105(5):106602. doi: 10.1016/j.psj.2026.106602 (PMC12925552; doi:10.1016/j.psj.2026.106602)

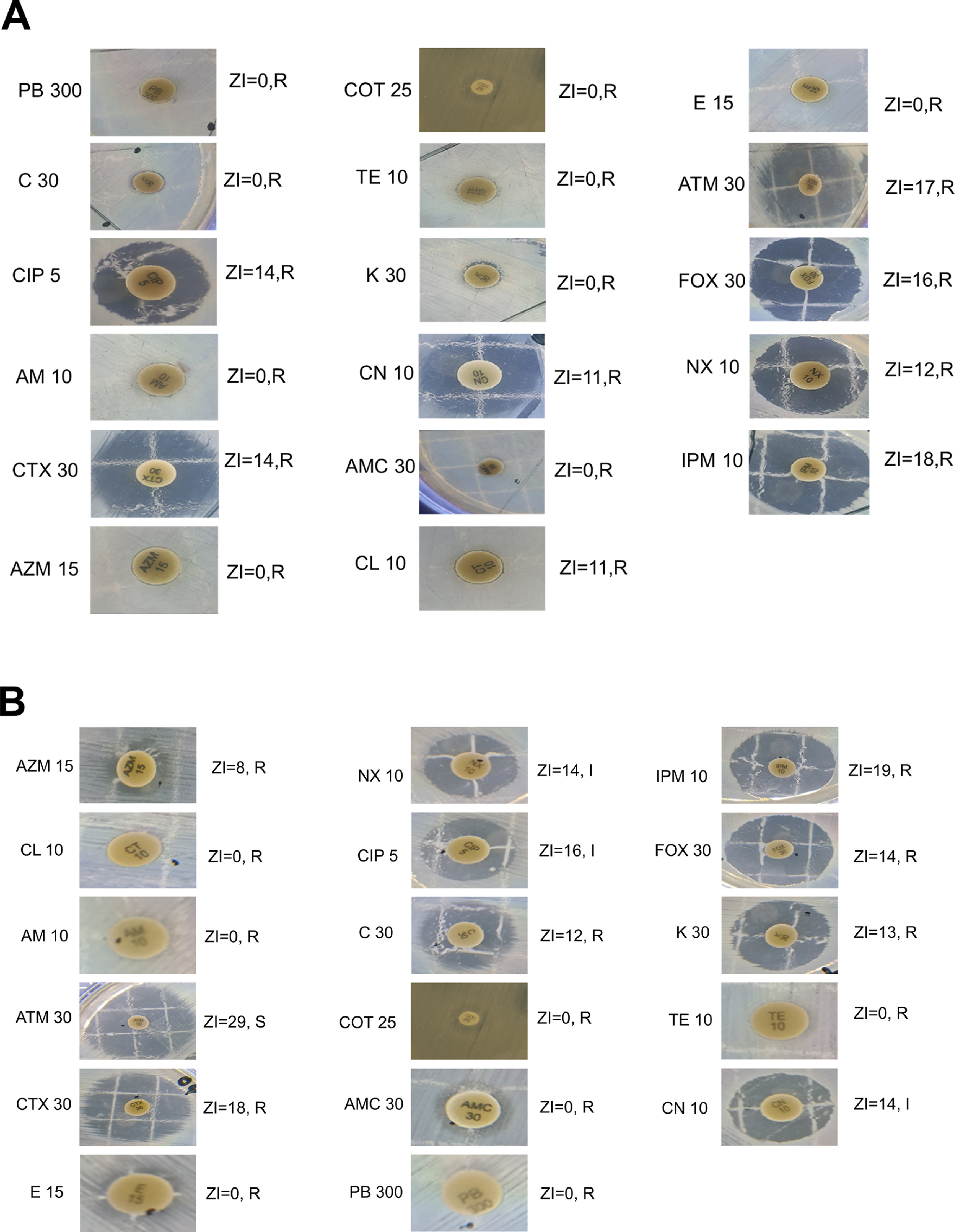

Supplement: Supplementary file 2 — List of Supplementary Figures Supplementary Figure S1: Antibiogram test of (A) P. stuartii ps_nstu_001 and (B) P. stuartii ps_nstu_002 by Kirby-Bauer disk diffusion method using 17 antibiotics. (Ampicillin (AMP 25), Amoxicillin-clavulanic acid (AMC 30), Cefotaxime (CTX 30), Cefoxitin (CX 30), Ciprofloxacin (CIP 5), Norfloxacin (NX 10), Aztreonam (ATM 30), Gentamicin (GEN 10) and Kanamycin (K 30), Azithromycin (AZM 30), Erythromycin (E 10), Imipenem (IMP 10), Chloramphenicol (C 30), sulfonamides-Trimethoprim-Sulfamethoxazole (COT 25), Tetracycline (TE 30), Colistin (CL 10), and Polymyxin B (PB 300), (ZI= Zone of Inhibition in mm, S= sensitive, R= resistant, Zone of inhibition was interpreted according to CLSI, 2018). [file mmc2.jpg]

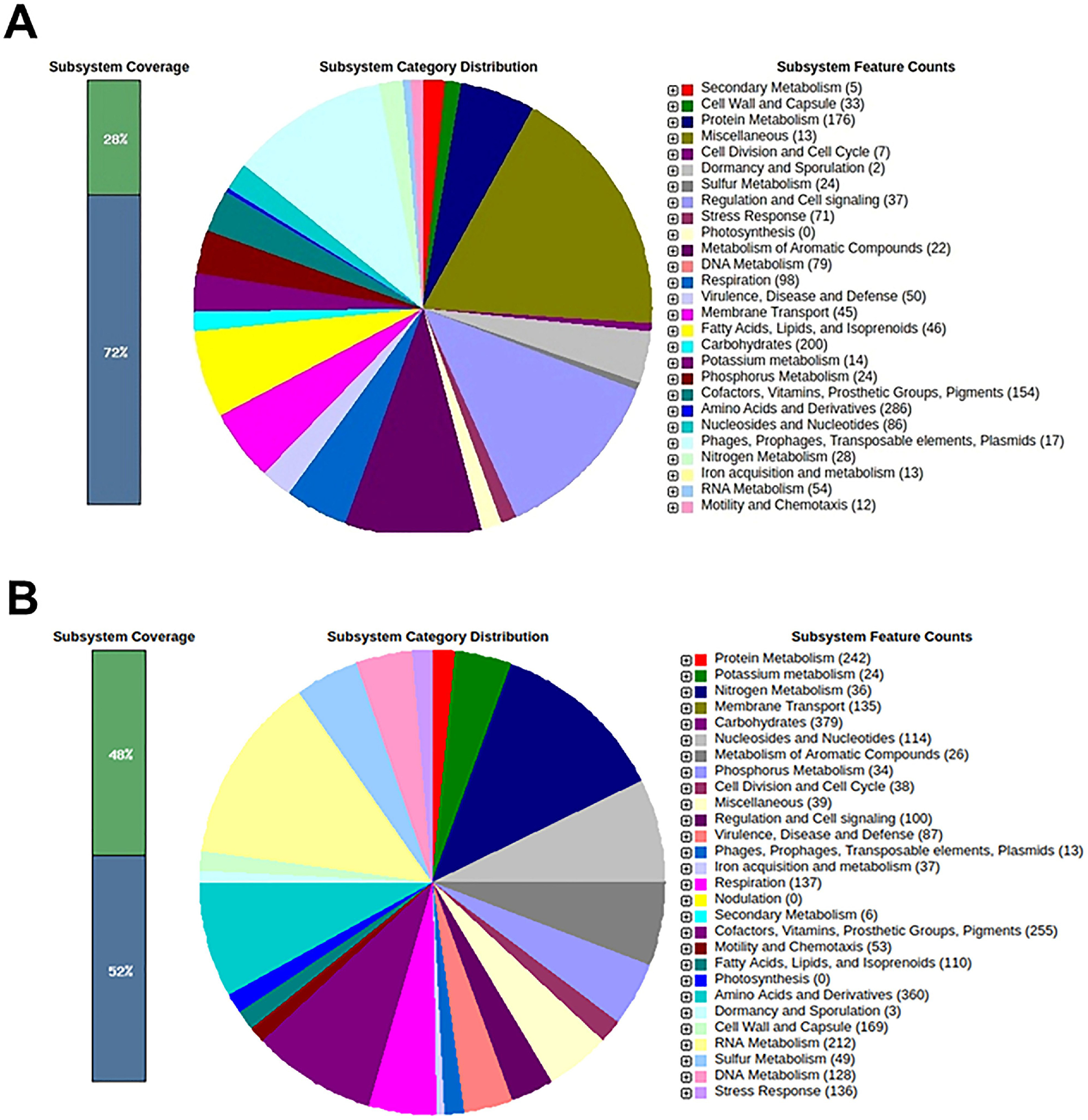

Supplement: Supplementary file 3 — Supplementary Figure S2: The subsystem annotation of (A) P. stuartii ps_nstu_001 genome and (B) P. stuartii ps_nstu_002 genome. [file mmc3.jpg]
